# Spatio-temporal epidemiology of animal and human rabies in northern South Africa between 1998 and 2017

**DOI:** 10.1371/journal.pntd.0010464

**Published:** 2022-07-29

**Authors:** Kgaogelo Mogano, Toru Suzuki, Debrah Mohale, Baby Phahladira, Ernest Ngoepe, Yusuke Kamata, George Chirima, Claude Sabeta, Kohei Makita

**Affiliations:** 1 Department of Geography, Geoinformatics and Meteorology, University of Pretoria, Pretoria, Republic of South Africa; 2 Soil, Climate and Water Institute, Agricultural Research Council, Arcadia, Pretoria, Republic of South Africa; 3 Department of Environmental and Symbiotic Science, College of Agriculture, Food and Environmental Sciences, Rakuno Gakuen University, Ebetsu, Japan; 4 Onderstepoort Veterinary Research (OVR), Agricultural Research Council, Onderstepoort, Pretoria, Republic of South Africa; 5 Department of Veterinary Medicine, School of Veterinary Medicine, Rakuno Gakuen University, Ebetsu, Japan; 6 Department of Veterinary Tropical Diseases, Faculty of Veterinary Sciences, University of Pretoria, Onderstepoort, Pretoria, Republic of South Africa; University of Surrey, UNITED KINGDOM

## Abstract

**Background:**

Rabies is a fatal zoonotic disease that is maintained in domestic dogs and wildlife populations in the Republic of South Africa. A retrospective study was conducted to improve understanding of the dynamics of rabies in humans, domestic dogs, and wildlife species, in relation to the ecology for three northern provinces of South Africa (Limpopo, Mpumalanga, and North-West) between 1998 and 2017.

**Methods:**

A descriptive epidemiology study was conducted for human and animal rabies. Dog rabies cases were analyzed using spatio-temporal scan statistics. The reproductive number (*R*_t_) was estimated for the identified disease clusters. A phylogenetic tree was constructed based on the genome sequences of rabies viruses isolated from dogs, jackals, and an African civet, and Bayesian evolutionary analysis using a strict time clock model. Several ecological and socio-economic variables associated with dog rabies were modeled using univariate analyses with zero-inflated negative binomial regression and multivariable spatial analyses using the integrated nested Laplace approximation for two time periods: 1998–2002 and 2008–2012.

**Results:**

Human rabies cases increased in 2006 following an increase in dog rabies cases; however, the human cases declined in the next year while dog rabies cases fluctuated. Ten disease clusters of dog rabies were identified, and utilizing the phylogenetic tree, the dynamics of animal rabies over 20 years was elucidated. In 2006, a virus strain that re-emerged in eastern Limpopo Province caused the large and persistent dog rabies outbreaks in Limpopo and Mpumalanga Provinces. Several clusters included a rabies virus variant maintained in jackals in Limpopo Province, and the other variant in dogs widely distributed. The widely distributed variant maintained in jackal populations in North-West Province caused an outbreak in dogs in 2014. The *R*_t_ was high when the disease clusters were associated with either multiple virus strains or multiple animal species. High-risk areas included Limpopo and Mpumalanga Provinces characterized by woodlands and high temperatures and precipitation.

**Conclusion:**

Canine rabies was maintained mainly in dog populations but was also associated with jackal species. Rural communities in Limpopo and Mpumalanga Provinces were at high risk of canine rabies originating from dogs.

## Author summary

Rabies is a fatal zoonotic disease transmitted primarily via bites by animals infected with the rabies virus. The majority of human cases occur due to the exposure to infected domestic dogs. In the Republic of South Africa, rabies is endemic, and domestic dogs, mongooses, bat-eared foxes, and jackals are the main reservoir hosts. Rabies control efforts typically target domestic dog populations, as it is difficult to monitor and control in wildlife species. Jackals are known to sustain infection cycles with domestic dogs. This study was conducted to improve understanding of the spatial and temporal dynamics of rabies in human, domestic dogs, and wildlife, in relation to ecology and socio-economics in northern South Africa. Human cases occurred where disease clusters in dogs were identified. Large size outbreaks in dogs were caused by dog rabies virus variants, but disease clusters included multiple virus variants in multiple host species. Rural communities in Limpopo and Mpumalanga Provinces, characterized by woodlands and high temperatures and precipitation, were at high risk of canine rabies originating from dogs.

## Introduction

Rabies is a fatal zoonotic disease caused by viruses of the *Rhabdoviridae* family (*Lyssavirus* genus and order of the *Mononegavirales*) [1,2]. Every year, at least 59,000 human rabies deaths are estimated to occur throughout the world [3], with the exception of nation islands. The vast majority of these human deaths (≥98%) are dog-mediated and occur in rural areas of Africa and Asia [3–5]. Although safe and efficacious anti-rabies vaccines are available for post-exposure management, unfortunately, rabies is a neglected disease, enabling human rabies to persist in the low economic countries of the world [6,7]. The most cost-effective way to control rabies is via parenteral vaccination of dogs [8], including stray and unowned dogs. The World Organisation for Animal Health (OIE) Rabies Reference Laboratories, the OIE Committee on Dog Rabies Control, and the World Health Organization advocate that oral rabies vaccination should play a vital role in synergy with parenteral dog vaccination to reach unvaccinated and stray dogs towards the global elimination of dog-mediated human rabies deaths by 2030 [9].

The epidemiology of rabies in South Africa is complex due to the presence of both domestic dog and wildlife rabies cycles [10–12]. In this context, the rabies virus is maintained in South Africa via complex epizootic cycles that involve a variety of different host species [13,14]. Apart from domestic dogs, wildlife species such as black-backed jackals (*Canis mesomelas*), bat-eared foxes (*Otocyon megalotis*), and the yellow mongooses (*Cynictis penicillata*) contribute to rabies epizootiology. The first two carnivore species maintain the canid rabies biotype, and the third maintains the mongoose rabies biotype [12,14,15], formerly known as the viverrid biotype [14]. In northern South Africa, however, the domestic dog and black-backed jackals both maintain the canid rabies biotype, an opportunistic lyssavirus variant that easily crosses species barriers [16,17].

**Fig 1:**
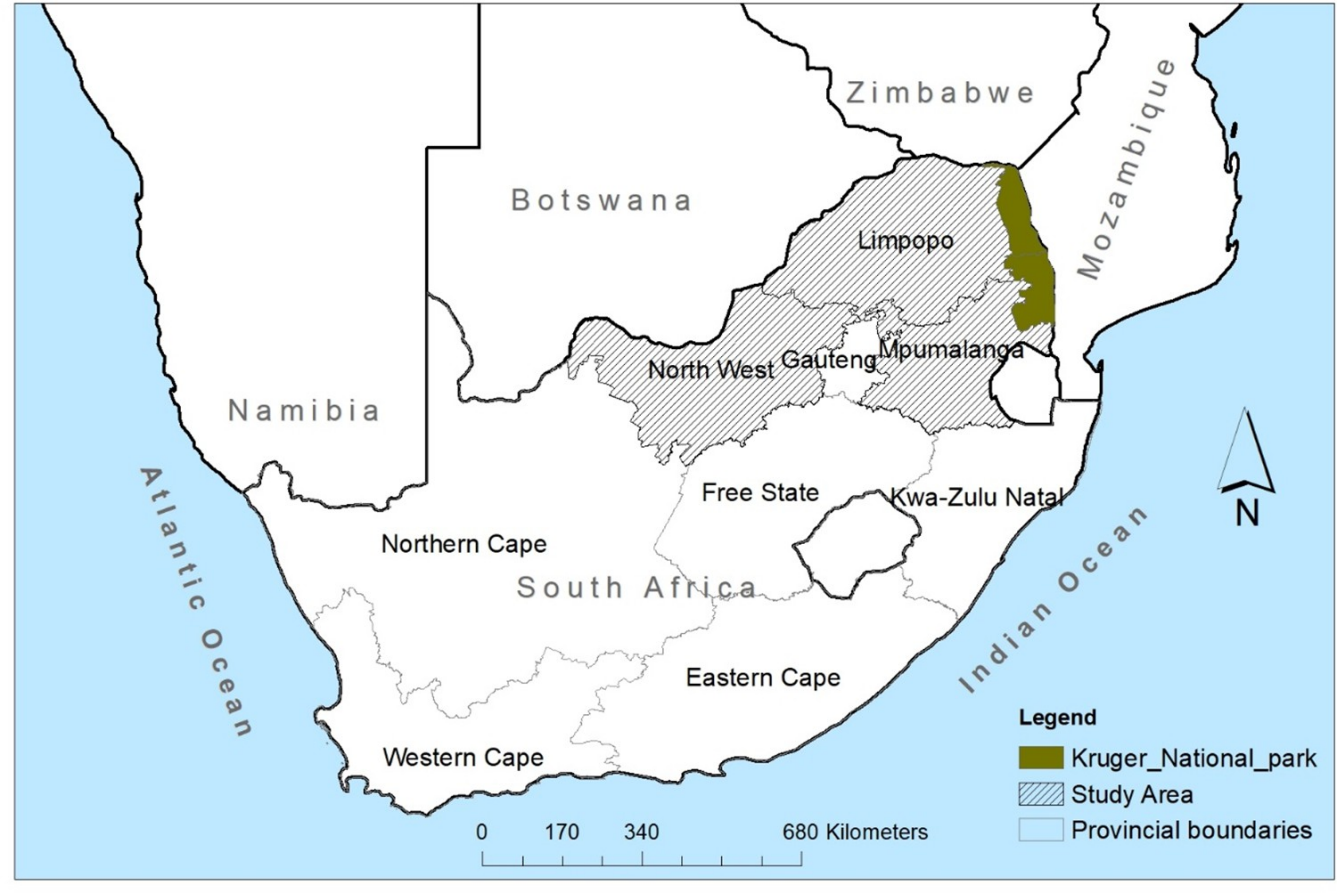
Map showing the provinces of South Africa and study areas. < https://dataportal-mdb-sa.opendata.arcgis.com/>.

Historically, the first rabies case in South Africa was reported in 1893 in the Port Elizabeth area, apparently introduced by a Terrier dog imported from England [18]. In the late 1950s, rabies was reported in Limpopo Province (Fig 1) apparently brought in by dogs from Angola, and also spread through Namibia and Botswana [18]. Today, dog rabies occurs in many parts of the country and is endemic in many communal areas in the Eastern Cape, Limpopo, Mpumalanga, and Kwazulu-Natal Provinces [11]. In the drier western regions of the country, wildlife rabies is prevalent, with the bat-eared fox being the maintenance host for canid rabies [15]. Recent research suggests that the aardwolf may also play a role in canid rabies epizootics in western South Africa [19]. Over the last two decades, rabies has emerged in areas where it had not been diagnosed before, demonstrating a geographic expansion of the disease and increase in the number of host species involved [20]. In light of the above, it is critical to determine the environmental conditions under which spill-over from wildlife to domestic species and the reverse frequently occurs [21].

Several molecular epidemiological studies have been conducted to elucidate the transmission dynamics of rabies in northern South Africa. The dog-mediated rabies outbreaks in Limpopo Province in 2005 and 2006 resulted in 21 confirmed, 4 probable, and 5 possible human deaths [22]. It was further shown that the canid rabies viruses involved in the 2005 rabies outbreak were closely related to those originating from black-backed jackals from southern Zimbabwe [11,22], demonstrating the transboundary nature of rabies. A second molecular study demonstrated the re-emergence of canid rabies in dogs in Mpumalanga Province in 2008, which was caused by a rabies variant similar to one that initiated previous outbreaks in the province. Wild dog (*Lycaon pictus*) rabies outbreaks occurred in 2000 and 2014 in the Madikwe Game Reserve in the North West Province; these outbreaks were linked to a wildlife source, probably black-backed jackals [23,24].

The epidemiology of rabies in northern South Africa has not been systematically and comprehensively analyzed. As wildlife species play a pivotal role in maintaining rabies in South Africa, understanding the spatial and temporal patterns of dog and wildlife rabies and their relationships with the ecosystem dynamics is crucial to predict and manage disease spread [25,26]. Moreover, socio-economic factors can contribute to making certain geographical areas more susceptible to the emergence of zoonotic infectious diseases [27,28]. In the case of rabies, socio-economic factors reportedly affect the conduct of dog rabies vaccination and dog management practices [29].

In this study, we conducted spatio-temporal analyses of rabies in northern South Africa using historical and laboratory-confirmed rabies cases from 1998 to 2017. The objectives were to (1) examine geographic patterns and characterize rabies trends over the 20-year period; (2) identify spatio-temporal clusters of animal rabies cases; and (3) evaluate the nature and extent of ecological factors that could facilitate such patterns. The ultimate aim of the study was to enhance understanding of the molecular evolutionary dynamics of rabies within the spatio-temporal clusters. The findings of the study will hopefully help us elucidate the mechanism of rabies dynamics and identify rabies hot spots, thus enabling more-effective resource allocation for targeted rabies control in South Africa.

## Methods

### Study area

The study area comprised three provinces located in the northern part of South Africa, namely, Limpopo, Mpumalanga, and North-West Provinces (Fig 1). Geographically, Limpopo Province is located at 23°24’00"S; 29°24’36"E, Mpumalanga Province at 25°33’36"S; 30°31’12"E, and North-West Province at 26°39’36"S; 25°16’48"E. Both Limpopo and Mpumalanga Provinces share borders with the Kruger National Park (KNP). The climate is variable, with temperate and subtropical areas, and most of the human population lives in rural villages and rely on subsistence farming of maize and rearing livestock.

### Data collection

Brain specimens from animals with suspected rabies are routinely submitted to the Rabies Reference Laboratory at the Onderstepoort Veterinary Research (OVR) in Pretoria, South Africa, to rule out or confirm rabies infection. Data for all rabies-suspect animals used in the present study and during the period 1998–2017 were retrieved from OVR. All the data used were historical sequences reported elsewhere and are publicly available in Genbank. These records included the following epidemiological information: animal species of origin, date of submission, date of diagnosis, location of rabies-suspect animal, rabies diagnosis result, province of origin, state veterinarian location, laboratory number and geographic coordinates. The coordinates were assigned based on the locality stated on the specimen submission forms or the locality of the state veterinary clinic if the original location was not indicated. All confirmed cases were recorded spatially in decimal degrees east and decimal degrees south. The dataset was created according to the boundaries of the Limpopo, Mpumalanga, and North-West Provinces using an Excel spreadsheet (Microsoft Office).

Human rabies data were obtained from the National Institute of Communicable Disease (NICD) in Johannesburg. The data included types of animal exposure, location, date of exposure, and number of post-exposure prophylaxis doses administered and was acquired from local clinics and hospitals. The NICD of the National Health Laboratory is exclusively mandated to test for human rabies in South Africa. The data of human rabies cases used in this study have been already published elsewhere [30].

The provincial, local municipality, and ward boundaries corresponding to the study areas were downloaded from the Municipal Demarcation Board, Centurion, South Africa (https://dataportal-mdb-sa.opendata.arcgis.com/). The administrative boundaries along with the rabies cases were projected into a Universal Transverse Mercator-WSG84 coordinate system. Population census data for 2001 and 2011 by local municipality were supplied by Statistics South Africa (Stats SA). These census data enumerated populations up to the ward level, which is the smallest unit. The human population was classified according to those living in urban versus rural areas. According to Stats SA, urban areas include formal and informal settlements, whereas rural settlements include farms and tribal authorities. Human population data based on community surveys in 2006 and 2016 enumerated populations at the local municipality level but not ward level.

Monthly temperature and rainfall data were obtained from WorldClim, version 2 [31]. From the WorldClim data, mean temperatures of driest quarter (BIO9) and precipitation of driest quarter (BIO17) were used for the analyses. Land cover data for 2001 and 2011 were obtained in a raster grid format from Global Land Cover by National Mapping Organization, version 2 [32]. Human footprint data for 1995–2004 and 2009 were obtained from Last of the Wild, v2 [33] and v3 [34].

### Data analysis

A descriptive epidemiology was conducted for the summary of rabies cases per province and domestic animal and wildlife host species between 1998 and 2017, and epidemic curves in dogs, wildlife, and humans were generated. The positivity rates in the three provinces were compared using multiple comparisons with the Holm adjustment method. The geographical locations of laboratory confirmed rabies cases were indicated together with changes in land use. To show spatial relationships between animal and human rabies cases, choropleth maps of dog rabies cases were produced, with the locations of human victims indicating the source animals for four time series: 1998 to 2002, 2003 to 2007, 2008 to 2012, and 2013 to 2017.

Kulldorff’s space-time scan statistics was performed using the canine rabies data for the four abovementioned time series between January 1998 and December 2017 using SaTScan, version 9.4.4 [35]. A discrete Poisson model was selected, and the maximum spatial cluster size was arbitrarily defined as 50% of the population at risk, with a circular window with a radius of 100 km. The time unit was set as month. The daily number of canine cases submitted to the laboratory for testing in the local municipality in which the cases occurred and the dates of the submissions were used. Geographical centroids calculated for the local municipalities using ArcMap (ESRI, USA) were used as the geographical representation of the locations of dog rabies occurrence. The dog populations in the municipalities were estimated using the human population data and published human-dog ratios: 21.0 humans vs. 1 dog in urban and 7.7 humans vs. 1 dog in rural areas [3,36]. For 2001 and 2011, the dog populations were estimated at the ward level and aggregated at the municipality level. For 2006 and 2016, as human populations were enumerated at the local municipality level, the proportions of urban and rural areas occupancy were calculated using land cover data, and local municipality human populations were allocated to respective urban and rural areas based on the population density ratios in 2001 and 2011, respectively. The dog populations in urban and rural areas in the local municipalities were calculated and aggregated to determine local municipality dog populations. The level of each detected cluster was considered statistically significant when the *p* value was <0.05 as evaluated using 999 Monte Carlo Simulation.

The effective reproduction number (*R*_t_), the number of secondary cases caused by an infected individual at the time of observation was calculated using Eq 1 for each significant cluster detected [37]. In the Republic of South Africa, accurate dog rabies vaccination records are not easily accessible. The proportion of vaccinated dogs in each study area was thus unknown; therefore, the *R*_t_ calculated here is influenced by the unknown proportion of dogs immunized.

$$R_{t}=1/\sum_{t=0}^{\infty }T_{s∙t}\lambda^{-t},$$ (1)

where *T*_*s*∙*t*_ is the distribution of the serial interval reported from a study in Tanzania [38], and *λ*^−*t*^ is the growth rate at time *t* (by week). To calculate the growth rate, after the exponential growth portion of the accumulated cases was selected, a generalized linear model with Poisson error was performed using weekly aggregated dog rabies data.

To elucidate the molecular evolutionary dynamics within detected clusters, partial genome sequences of the glycoprotein gene and intergenic regions of previously characterized dog and wildlife rabies viruses associated with the spatio-temporal clusters were selected (S1 Table). The selection criteria were: dog rabies samples from (1) the central part of the spatial cluster, (2) the time of high dog rabies incidence within the cluster, and (3) any wildlife rabies sample(s) within the clusters. For dog rabies, several viruses were selected from the cluster when the outbreak persisted for a long period (longer than a year). The nucleotide sequences of the cytoplasmic domain of the glycoprotein and G-L intergenic region of the viruses were aligned using ClustalW (MEGA X software package) and saved as a NEXUS file. Firstly, the nexus file of the previously characterized rabies viruses was subjected to Maximum Likelihood Analysis and then subsequently loaded in Bayesian Evolutionary Analysis Utility version 2.6.0 [39], and a BEAST XML file was created with the settings of the year of sample submission as dates, HKY for DNA substitutions, and a strict clock model. A Bayesian Markov Chain Monte Carlo simulation was run for 2 million iterations using BEAST2, version 2.6.0 [39], and simulation results were stored every 5000 iterations. The posterior distributions were subsequently inspected using Tracer software (version 1.7.1) to ensure adequate mixing and coverage. The phylogenetic tree was visualized using FigTree, version 1.4.4 [39].

To identify ecological factors associated with the risk of dog rabies, two-time periods (1998–2002 and 2008–2012) were selected, as detailed demographic data were available in 2001 and 2011 from the concluded national census. The 20 land cover types in Global Land Cover were reclassified into six types: (1) woodland, (2) arid, (3) farmland, (4) bare, (5) urban, and (6) water (S1 Fig). The area of each land cover type was extracted by local municipality (Spatial Analyst, ArcMap). Correlations among five of the reclassified land cover types, excluding water, were then analyzed for each time period. Using principal component analysis (PCA), a set of possibly correlated land cover types was converted into a set of linearly uncorrelated variables called principal components (PCs). Three PCs each for which the standard deviation was >1.0 were selected for period 1 (2001 data for 1998–2002) and period 2 (2011 data for 2008–2012). Mean temperature and precipitation for the driest quarter were selected as two bioclimatic variables that could be associated with rabies incidence.

To test the hypothesis that a socio-economic factor, poverty, is a driver of dog rabies, a cut-off threshold of population density was selected to generate a layer of low-income residential areas. In October 2019, field work was conducted in urban areas of Gauteng Province, which included residential areas of apparently high income (Waterkloof, Brooklyn, and Menlyn suburbs) and low income (Mamelodi Township). No free-roaming dogs were observed in the high-income residential areas, whereas free-roaming dogs were observed in the low-income areas. The boundaries of both the low- and high-income residential areas were demarcated using Google Earth. The population density for each residential area was calculated using estimated total population (2011 census) for each residential area divided by the total surface area. The population density was 1139/km^2^ for the high-income area and 4844/km^2^ for the low-income area. The cut-off value for the low-income residential area was set at >4000/km^2^. The other socio-economic factors included in this study were dog population density and human footprint (S2 Fig for the years 1995–2004, and S3 Fig for 2009).

Univariable analyses were conducted to examine the association between rabies incidence and the abovementioned PCAs, climatic factors, and socio-economic factors using zero-inflated negative binomial (ZINB) regression with the offset of logarithm of area of local municipalities in the R pscl package [40] of statistical software R, version 4.0.1 [41]. Only variables that were significant at a liberal *p* < 0.1 were retained for further analysis. Collinearity (rho >0.6) was assessed for variables with potentially similar ecological meaning to avoid including them in the same multivariable model. Spatial models were compared among the convolution model, Besag’s spatial model, and proper version of Besag’s spatial model based on the lowest value of the deviance information criterion (DIC) and widely applicable information criterion (WAIC). Multivariable integrated nested Laplace approximation (INLA) convolution models were performed for all possible combinations of explanatory variables selected in the univariable ZINB regression analyses, using the R-INLA package, version 19.09.03 [42]. The KNP was a special case in that there are no human communities in the area except administrative staff and family, and those enumerating dog rabies cases from surrounding communities at the Mpumalanga Provincial State Veterinary Services Office: Bushbuckridge East- Orpen (SV BBR E O) [43]. Therefore, this ecological risk factor analysis was performed with both datasets which included and excluded the KNP. The best model was selected based on DIC and WAIC. These analyses were conducted for both time periods. Mapping and spatial-temporal analyses were performed using ArcMap, version 10.3.

## Results

### Descriptive epidemiology

**Fig 2:**
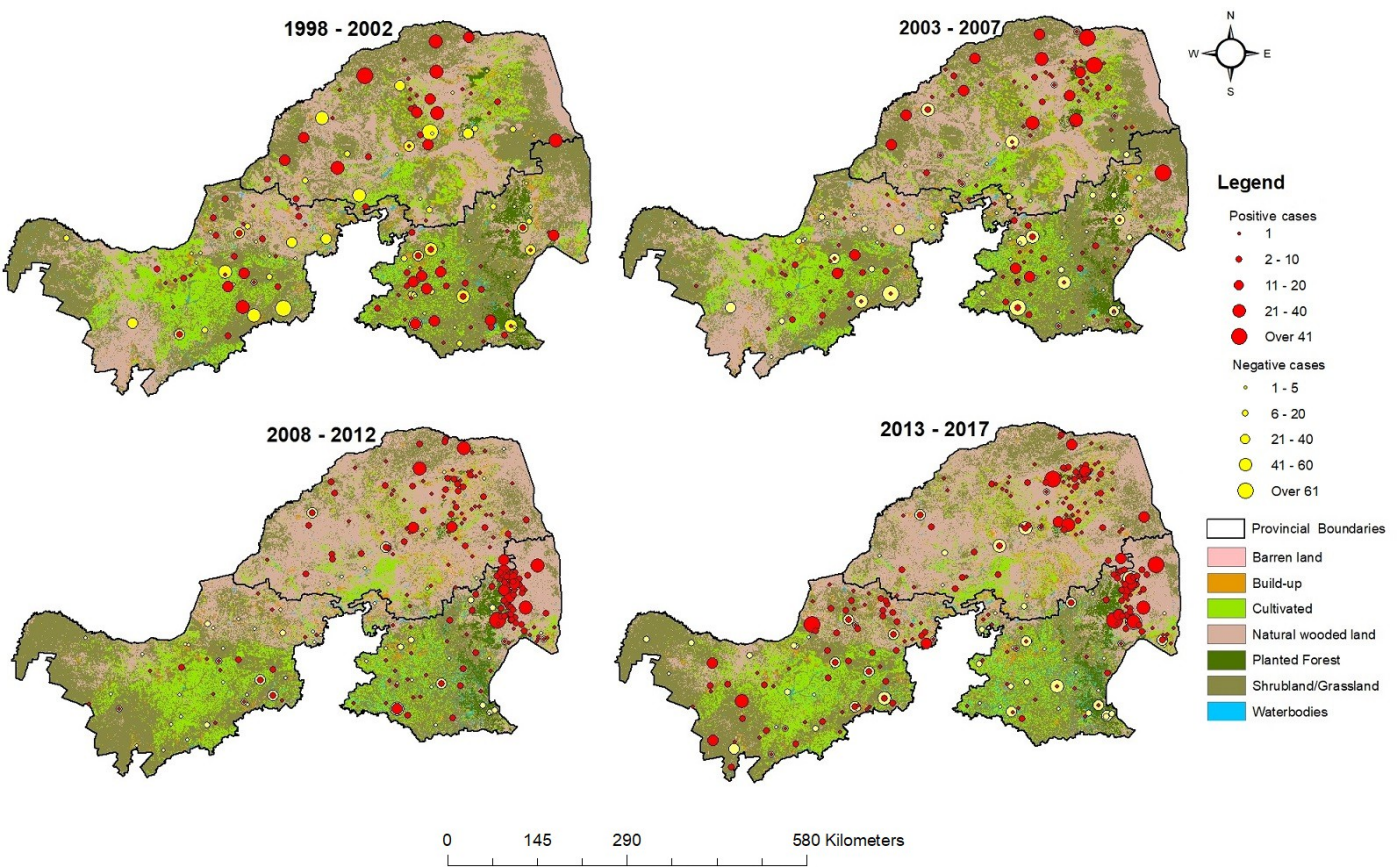
Spatial distributions of positively and negatively diagnosed animals between 1998 and 2017, and changes in land use over time. < https://www.dffe.gov.za>.

During the period 1998 to 2017, a total of 8,464 animal brain tissue samples were tested, of which 30.7% (2,605/8,464) were confirmed positive for rabies. The positivity rate was highest in Limpopo (43.2%, 1,075/2,490, *p*<0.001) compared with the other two provinces, followed by Mpumalanga (27.5%, 1,130/4,113) and North-West Provinces (24.6%, 458/1,861, *p* = 0.022; compared with Mpumalanga) in multiple comparisons. Fig 2 shows the spatial distributions of animals tested and laboratory confirmed as positive or negative relative to changes in land use. The size of the dots show the relative number of diagnoses in the same location (local veterinary centers as the representative positions for the samples without location information). Rabies diagnosis efforts were carried out across the study areas, as demonstrated by the distribution of confirmed negative results. Mapping of localities with positive rabies tests revealed patchy concentrations in the study areas, with the pattern of some remaining constant and that of others changing. The overlapping of positive and negative diagnoses with high human footprint (S2 and S3 Figs) was observed in northeastern districts of both Limpopo and Mpumalanga Provinces (Fig 2). Changes in vegetation were the most apparent, with the replacement of grassland with naturally wooded land, particularly in Limpopo Province.

Among all positive animal rabies cases, domestic animals accounted for 78.5% (2,045/2,605) and wildlife 21.5% (560/2,605) (Table 1). The overall positivity rates were 33.8% in domestic animals and 30.8% in wildlife. Of the domestic animal rabies cases, dogs accounted for the majority of cases at 69.8% (1,428/2,045 cases), followed by cattle (22.9%, 469/2,045 cases). The wildlife species most commonly diagnosed with rabies were jackals (45.5%, 255/560 cases) and mongooses (43.3%, 242/560 cases).

**Table 1:** Number of animal specimens submitted to the laboratory and confirmed as positive, and positivity rates for domestic and wildlife species.

Species	Specimens submitted	Positive	Positive rates(%)
Domestic animals			
Dogs	3,841	1,428	37.2
Cattle	1,463	469	32.1
Cats	516	47	9.1
Sheep	54	14	25.9
Goats	97	58	59.8
Donkey/horses	50	22	44.0
Pigs	23	7	30.4
Sub-total	6,044	2,045	33.8
**Wildlife**			
Jackals	442	255	57.7
Mongoose	1,043	242	23.2
Bat eared foxes	65	12	18.5
Civet	19	7	36.8
Wild cats	26	2	7.7
Wild dogs	28	4	14.3
Other wildlife	797	38	4.8
Sub-total	2,420	560	23.1
Total	8,464	2,605	30.8

**Fig 3:**
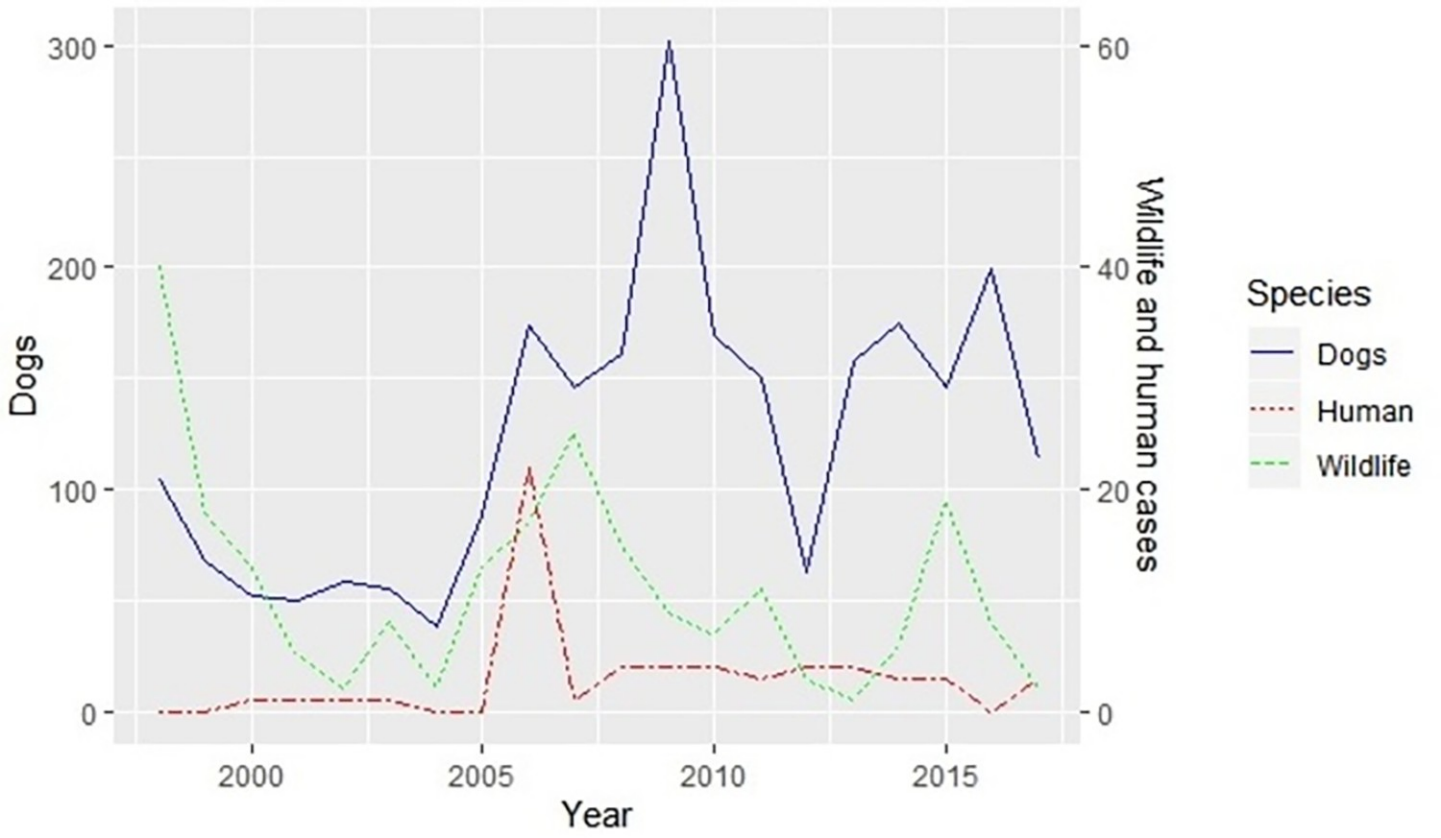
Temporal dynamics of human, dog, and wildlife rabies cases in northern South Africa between 1998 and 2017.

Fig 3 shows the temporal dynamics of human, dog, and wildlife rabies cases during the study period. During the first time period examined (1998–2002), a high frequency of confirmed rabies cases in wildlife species was observed, with a similar pattern in domestic dogs, and only a few human rabies cases. After this epidemic abated, however, a sharp increase in dog and wildlife rabies occurred in 2005 in Limpopo Province, with the highest number of human rabies cases reported in 2006. This epidemic then spread in dogs, while the number of human and wildlife cases declined in 2007 and 2008, respectively. Dog rabies epidemics occurred again in 2013 in Limpopo and Mpumalanga Provinces. The temporal increase in dog rabies cases was again followed by a concomitant increase in wildlife rabies cases. During the 2013 epidemic, the number of human rabies cases remained low. Over time, more dog rabies cases were reported than in humans or wildlife (see the different y-axis scale in Fig 3).

**Fig 4:**
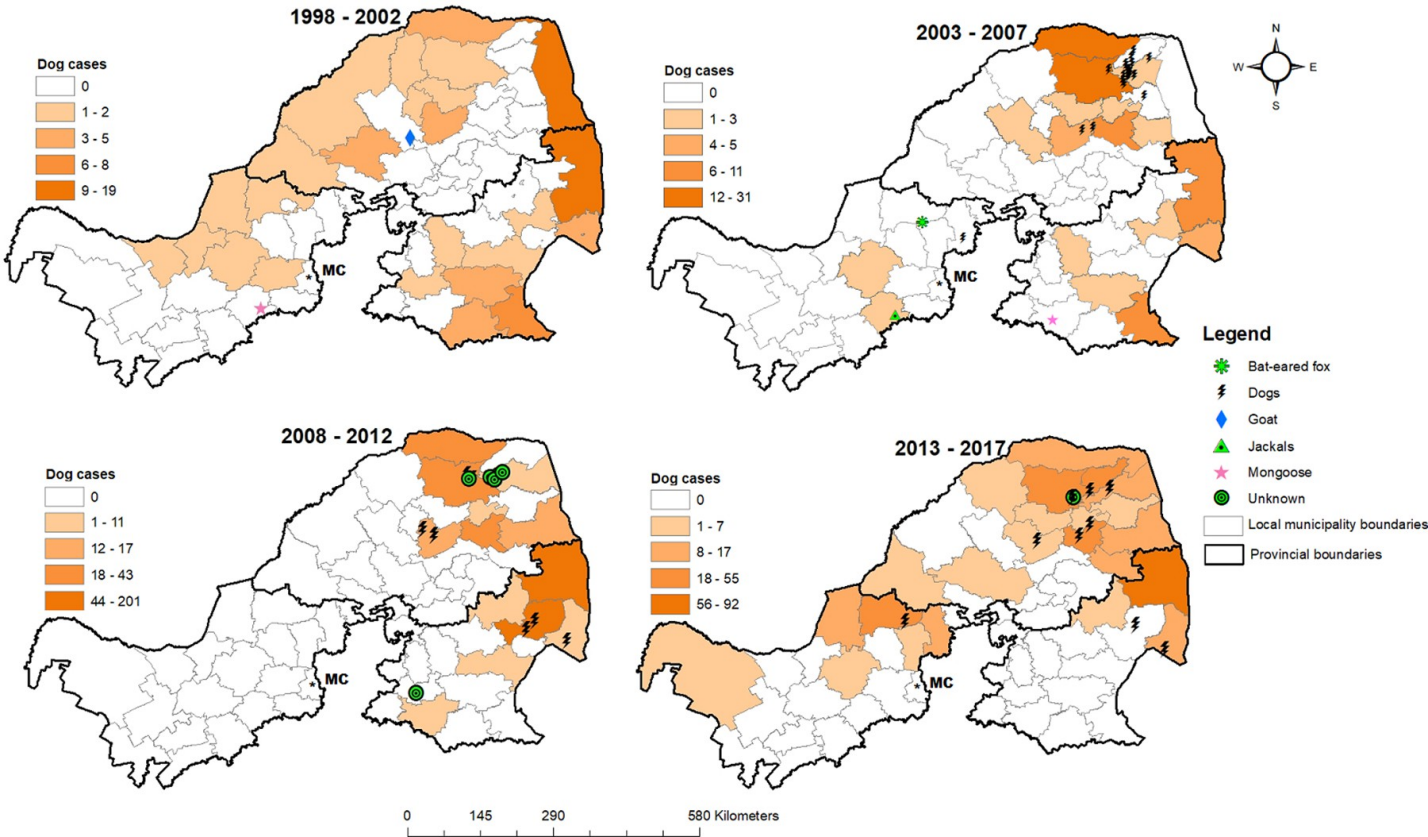
Choropleth maps of the distributions between 1998 and 2017 of dog rabies cases indicated per square kilometer by local municipality as a color gradient, and locations of human rabies victims shown by different colors and symbols indicating the source animals. < https://dataportal-mdb-sa.opendata.arcgis.com/>.

Fig 4 shows choropleth maps of the distributions of dog rabies cases by local municipality as a color gradient and locations of human rabies victims that succumbed (with different colors and symbols) with indication of the animal source across four time periods. In all four time periods, a high rate of dog rabies cases was observed in the northern and eastern parts of Limpopo Province and northeastern part of Mpumalanga Province, areas that are close to the KNP (Fig 1). North-West Province had the lowest number of dog rabies cases. In the 20 years under review, 57 laboratory-confirmed human rabies cases occurred in the study areas, with the highest number of victims from Limpopo (45), followed by Mpumalanga (7) and North-West (5) Provinces. Dogs accounted for 75.4% (43/57) of the human rabies cases. A geographical concentration in the human rabies cases associated with dog bites in Limpopo Province was observed in 2006, and the occurrence of human cases persisted in this province. Limpopo Province had multiple human rabies cases of unknown origin, particularly during the 2008–2012 period.

### Spatio-temporal analysis of animal rabies

**Fig 5:**
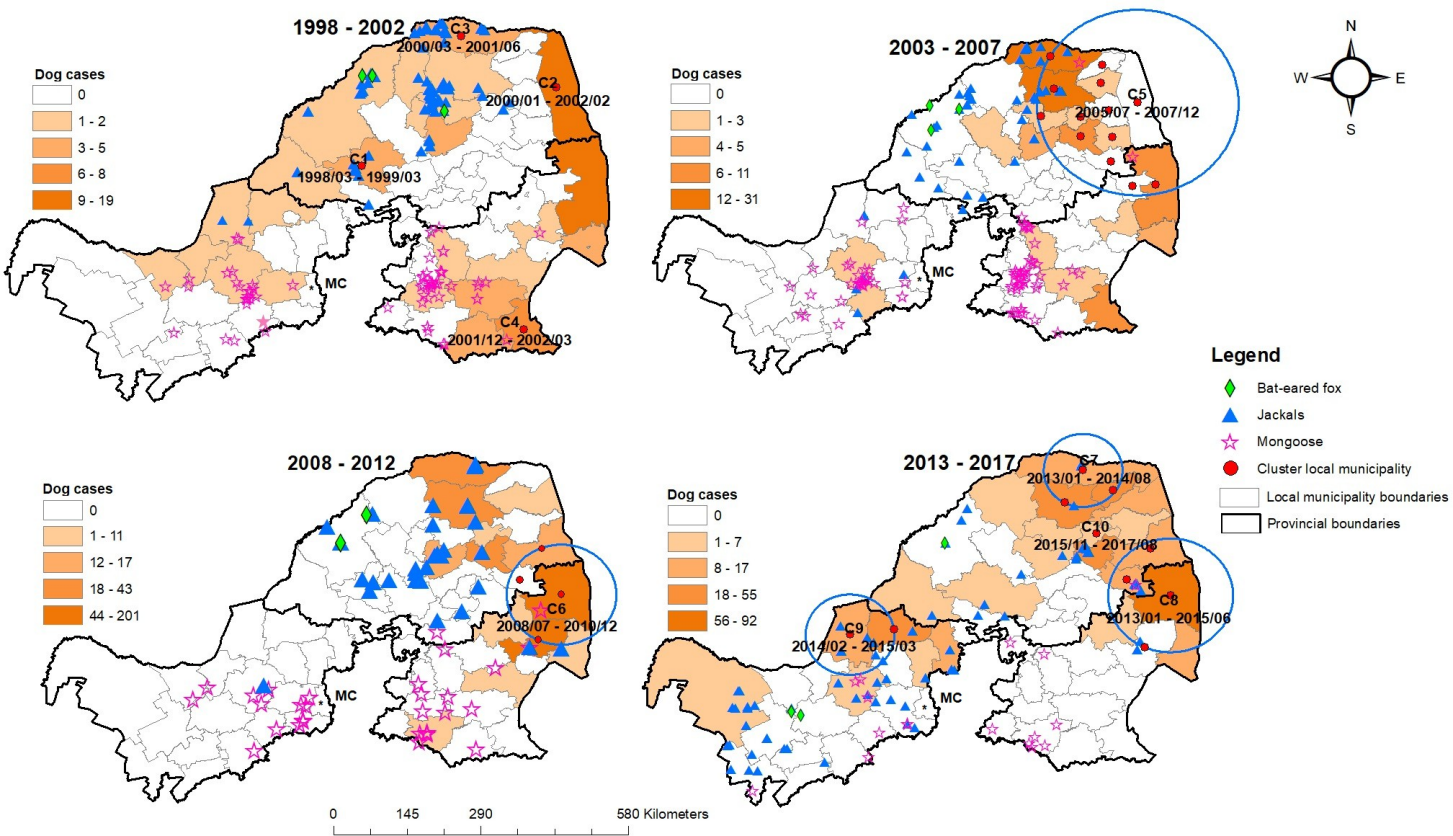
Spatio-temporal clusters of dog rabies in each time period and spatial distributions of wildlife rabies cases indicated on choropleth maps of dog rabies in four time periods between 1998 and 2017. Red dots indicate local municipalities affected by spatio-temporal clusters. MC denotes a local municipality of Gauteng Province, and was excluded from this study. < https://dataportal-mdb-sa.opendata.arcgis.com/>.

Space-time scan analysis identified 10 significant spatial clusters of dog rabies at the local municipality level between 1998 and 2017 (Fig 5, Table 2). Between 1998 and 2002, four significant clusters were detected involving small numbers of dog rabies cases in Limpopo and Mpumalanga Provinces (Fig 5, Table 2). These outbreaks were limited to the local municipalities; thus, the radii were not calculated. The fourth cluster (C4) in Mpumalanga Province in 2001–2002 exhibited a very steep (but not statistically significant) growth curve (slope = 0.14, se = 0.08, *p* = 0.095; note that the *p*-value refers to growth rather than spatial clustering), which resulted in a high mean *R*_t_ with wide confidence interval due to the low number of cases. This epidemic was successfully contained despite the high *R*_t_. In the second time series, 2003 to 2007, a large dog rabies epidemic involving 175 cases occurred in both Limpopo and Mpumalanga Provinces (C5, Fig 5, Table 2), highlighting the transboundary nature of rabies in this southern African country. In the third time series, 2008 to 2012, only one dog rabies epidemic occurred (C6), but it was the largest and longest and affected similar areas as C5 (Fig 5, Table 2), involving 331 cases. In the fourth time series, 2013 to 2017, four spatio-temporal clusters with high *R*_t_ values were observed. C9 was the only cluster that occurred in North-West Province, and this 2014–2015 epidemic was characterized by a high number of jackal rabies cases that expanded to the areas indicated in the time series (Fig 5). The size of cluster C10 was not calculated, as the epidemic remained limited to the local municipality in Limpopo Province.

**Table 2:** Time period, province, number of dog rabies cases, and size of the spatio-temporal clusters in South Africa and their reproduction number (*R*_t_).

ID	Time period	Province	Cases	Size (km)	*p*-value	*R*_t_
C1	1998/03-1999/03	Limpopo	4	-	0.035	1.58 (0.76–2.55)
C2	2000/01-2002/02	Mpumalanga	12	-	<0.001	1.43 (1.16–1.71)
C3	2000/03-2001/06	Limpopo	4	-	0.011	1.55 (0.73–2.53)
C4	2001/12-2002/03	Mpumalanga	5	-	<0.001	5.65 (0.47–13.6)
C5	2005/07-2007/12	Limpopo, Mpumalanga	175	184.2	<0.001	1.78 (1.74–1.83)
C6	2008/07-2010/12	Limpopo, Mpumalanga	331	94.8	<0.001	1.55 (1.50–1.60)
C7	2013/01-2014/08	Limpopo	54	68.2	0.003	2.64 (2.23–3.08)
C8	2013/01-2015/06	Limpopo, Mpumalanga	140	106.5	<0.001	1.91 (1.82–2.00)
C9	2014/02-2015/03	North West	20	75.3	0.002	3.65 (1.85–5.79)
C10	2015/11-2017/08	Limpopo	32	-	0.002	3.99 (2.17–6.11)

Geographically, the distributions of rabies in bat-eared foxes and mongooses were limited to the western part of Limpopo Province, North West province, and western part of Mpumalanga Province throughout the 20 years examined by the study (Fig 5). These distributions were not associated with spatio-temporal clusters of dog rabies. In contrast, the geographical distribution of jackal rabies almost always overlapped clusters of dog rabies, demonstrating the intrinsic association between the dog and jackal rabies cycles in this part of the country.

**Fig 6:**
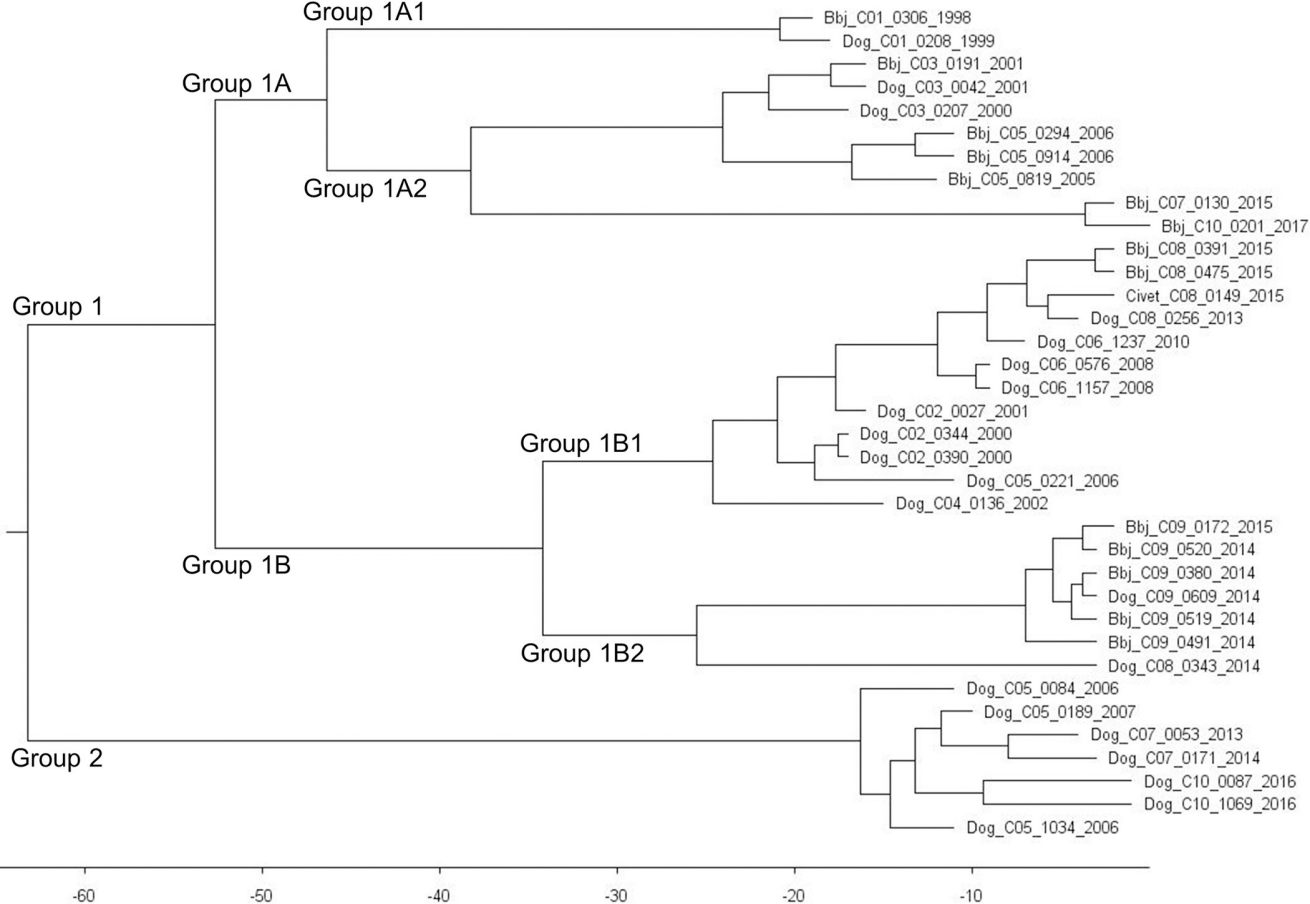
Phylogenetic tree of rabies viruses in dogs and wildlife in 10 spatio-temporal clusters of dog rabies between 1998 and 2017. The X-axis shows the estimated years of rabies virus evolution prior to 2017. The first block of the virus designation indicates the animal species: Dog, black-backed jackal (Bbj), or civet cat (Civet). The second block is the cluster identification, the third the virus code, followed by the year of sample submission.

Fig 6 shows a phylogenetic tree of rabies viruses originating from domestic dogs and wildlife species found within the 10 spatio-temporal clusters of dog rabies identified in the study. In total, 36 rabies viruses were used for the analysis. The X-axis shows the year of virus evolution prior to 2017. The topology of the phylogenetic tree (Fig 6) was similar to the maximum likelihood tree in MEGA (figure not shown). The 36 viruses were delineated into Groups 1 and 2. Group 1 viruses were split into Groups 1A and 1B respectively. Group 1A was composed of sub-clusters consisting of a mix of dog and jackal viruses, and two clusters exclusively composed of jackal rabies viruses from Limpopo Province. Group 1B on the other hand was composed of two clusters, Group 1B1 and 1B2 respectively. Group 1B1 comprised of dog viruses involved in dog rabies outbreaks in Mpumalanga Province (Nkomazi and Mbombela areas) and two black-backed jackal viruses originating from Makhado (Limpopo Province) showing a linkage of rabies cycles between Limpopo and Mpumalanga Provinces. Furthermore, a single rabies virus from an African civet demonstrated a linkage of dog and wildlife cycles. Group 1B2 was composed exclusively of black-backed jackal rabies viruses (from the North-West Province) and a dog virus (from the border between the North-West and Limpopo Provinces probably lending support to a recent outbreak in this wildlife species). Group 2 was associated with exclusively dog rabies outbreaks specifically in Sibasa, Tzaneen and Makhado (Limpopo Province) in the mid-2000s and between the years 2013–2016. The first introduction of rabies into South Africa according to Bayesian analysis occurred over 60 years ago.

### Ecological and socio-economic factors facilitating the occurrence of dog rabies outbreaks

In the 2001 dataset including the KNP, PC1 was characterized by a positive value in herbaceous (0.534) and woodlands (0.409) and negative value in shrub (-0.721), whereas PC2 was characterized by positive values in bare land (0.449) and urban areas (0.590) and a negative value in woodlands (-0.533). PC1 and PC2 had standard deviations greater than 1.0 and explain 64.4% of the variance (S2 Table). Univariable analyses using convolution modeling identified two significant factors: PC2 and precipitation in the driest quarter (Table 3).

**Table 3:** Univariable analysis results for dog rabies cases in South Africa between 1998 and 2002 including the Kruger National Park.

Variables	Estimate	Standard error	*p*-value
Dog population	-0.012	0.038	0.760
Human footprint	0.031	0.089	0.731
Densely-populated area	0.001	0.001	0.368
PC1	0.126	0.172	0.464
PC2	-0.412	0.218	0.058
Temperature	0.095	0.079	0.231
Precipitation	0.053	0.026	0.040

PC: Principal component

In the 2001 dataset excluding the KNP, PC1 was characterized by positive values in herbaceous (0.508), and woodland (0.448), and negative value in shrub (-0.721). PC2 was characterized by positive values of herbaceous (0.617), urban (0.595), and bare land (0.476), and negative values in woodland (-0.623). PC1 and PC2 explained 60.5% of the variance (S3 Table). Univariable analysis identified two same significant factors: PC2 and precipitation (Table 4), with the data including the KNP.

**Table 4:** Univariable analysis results for dog rabies cases in South Africa between 1998 and 2002 excluding the Kruger National Park.

Variables	Estimate	Standard error	*p*-value
Dog population	0.002	0.039	0.959
Human footprint	0.035	0.100	0.727
Densely-populated area	0.000	0.001	0.435
PC1	0.211	0.170	0.216
PC2	-0.452	0.22	0.043
Temperature	0.056	0.089	0.530
Precipitation	0.054	0.025	0.034

PC: Principal component

**Fig 7:**
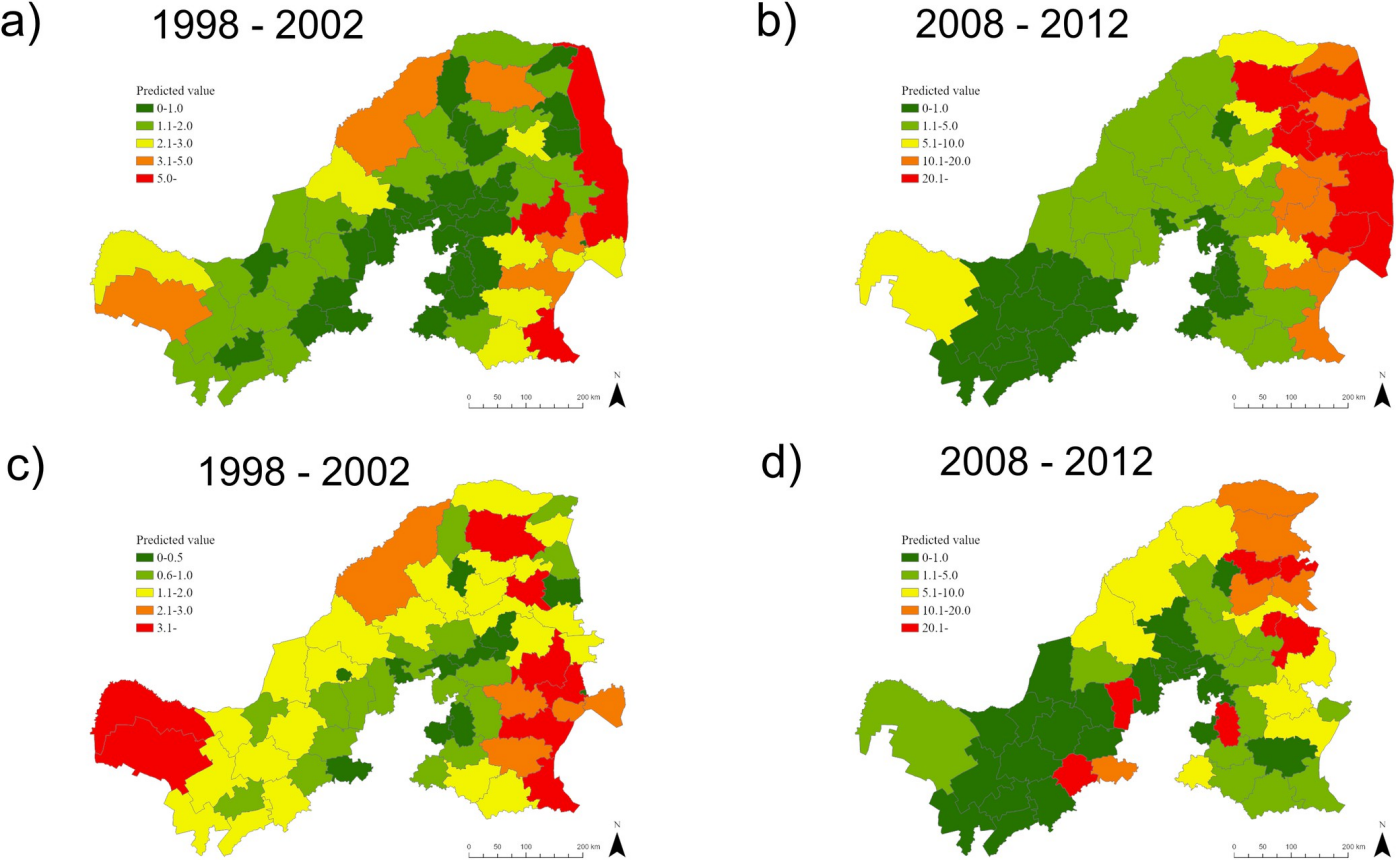
Predicted ecological risk of dog rabies. Panel a) the risk between 1998 and 2002 with the Kruger National Park (KNP); b) between 2008 and 2012 with the KNP; c) between 1998 and 2002 without the KNP; and d) between 2008 and 2012 without the KNP. Red municipalities had the highest rabies risk, whereas green areas had the lowest risk. < https://dataportal-mdb-sa.opendata.arcgis.com/>.

Table 5 shows the best multivariable models with the lowest DIC and WAIC for the datasets with and without the KNP between 1998 and 2002 (see S4 and S5 Tables for the model selection). For the multivariable analysis, both best models for the 1998–2002 period, with and without KNP, were explained by a negative value of PC2, suggesting richness in woodland, and higher precipitation in driest quarter (Table 5). These models showed high dog rabies risk in woodland areas in north and northeastern Limpopo Province, eastern Mpumalanga Province, and the western part of North-West Province, which supported dog rabies clusters (Fig 7A and 7C). In both models, spatially non-structured residual was greater than structured residual, suggesting risk of dog rabies can be well explained by ecological conditions, in addition to the influence of dog rabies occurrence in neighboring municipalities.

**Table 5:** Multivariable analysis results for INLA using a zero-inflated convolution models with negative binomial errors for predicting dog rabies cases in South Africa between 1998 and 2002 using datasets with and without Kruger National Park.

Variables	Dataset with KNP	Dataset without KNP
PC2	-0.336	-0.321
Precipitation	0.042	0.042
Spatially structured residual	1877.4	2204.7
Non-structured residual	1907.1	2339.2
WAIC	163.9	153.1

INLA: Integrated nested Laplace approximation; KNP: Kruger National Park; PC: Principal component; WAIC: Widely applicable information criterion

For the more recent ecological conditions in 2011, three PCs had standard deviations greater than 1.0 in the dataset with the KNP (S6 Table). PC1 was characterized by a positive value in arid areas (shrub, 0.708) and negative values in herbaceous (-0.547) and woodlands (-0.403). PC2 had high values in urban (0.592), bare (0.393), and farm land (0.264), and negative value in woodland (-0.536). PC3 was characterized by positive values in urban areas (0.144), bare land (0.573), and woodland (0.218).

The univariable analyses for the time series covering the period 2008 to 2012 in the dataset with the KNP had more significant variables than that of the 1998 to 2002 period (Table 6): dog population, PC2, temperature, and precipitation in driest quarter. Though not statistically significant, densely populated areas had a negative estimate, suggesting dog rabies is less likely to occur in low-income residential urban areas.

**Table 6:** Univariable analysis results for dog rabies cases in South Africa between 2008 and 2012 including the Kruger National Park.

Variables	Estimate	Standard error	*p*-value
Dog population	0.161	0.059	0.006
Human footprint	0.040	0.152	0.791
Densely-populated area	-0.001	0.001	0.116
PC1	-0.220	0.263	0.403
PC2	-0.899	0.232	<0.001
PC3	0.827	0.522	0.114
Temperature	0.396	0.098	<0.001
Precipitation	0.112	0.030	<0.001

PC: Principal component

Even in the 2011 dataset excluding the KNP, three PCs had standard deviations greater than 1.0 (S6 Table). PC1 was again characterized by a positive value in arid areas (shrub, 0.691) and negative values in herbaceous (-0.558) and woodlands (-0.423). PC2 had high value in woodland (0.465), and negative values in bare (-0.484), urban (-0.647), and herbaceous (-0.330). PC3 was characterized by positive values in farm land (0.815), and negative values in bare land (-0.496), and woodland (-0.264). The univariable analyses in the 2008–2012 dataset excluding the KNP had similar significant variables with the dataset including the KNP (Table 7): PC2, temperature, and precipitation in driest quarter. The sign of PC2 in Table 7 changed from Table 6, and so the signs of the values in woodland, bare land, and urban areas in PC2 changed.

**Table 7:** Univariable analysis results for dog rabies cases in South Africa between 2008 and 2012 excluding the Kruger National Park.

Variables	Estimate	Standard error	*p*-value
Dog population	0.09	0.05	0.09
Human footprint	0.008	0.153	0.960
Densely-populated area	-0.001	0.001	0.248
PC1	-0.111	0.282	0.694
PC2	0.528	0.276	0.056
PC3	-0.397	0.490	0.418
Temperature	0.291	0.115	0.011
Precipitation	0.064	0.031	0.038

PC: Principal component

The best multivariable model for the 2008–2012 dataset with the KNP was explained by high temperature and precipitation in the driest quarter (Table 8). All the multivariable models compared are listed in S8 Table. The best multivariable model in the dataset without the KNP included more variables: dog population and PC2 (all the models are listed in S9 Table). However, the sign of both these variables were different from univariable analyses. Both models included temperature and precipitation, and maps identified high risk areas in north and northeastern Limpopo Province (Fig 7B and 7D). In the risk map for 2008–2012 excluding the KNP, the risk in eastern Mpumalanga was estimated lower than the model with the KNP, and several districts surrounding Gauteng Province had high risk.

**Table 8:** Multivariable analysis results for INLA using a zero-inflated convolution models with negative binomial errors for predicting dog rabies cases in South Africa between 2008 and 2012 using datasets with and without Kruger National Park.

Variables	Dataset with KNP	Dataset without KNP
Dog population	-	-0.196
PC2	-	-2.189
Temperature	0.417	1.171
Precipitation	0.124	0.277
Spatially structured residual	1858.3	1866.7
Non-structured residual	1904.7	1764.2
WAIC	257.1	193.6

INLA: Integrated nested Laplace approximation; KNP: Kruger National Park; PC: Principal component; WAIC: Widely applicable information criterion

## Discussion

This study explored the dynamics of human and animal rabies in northern South Africa between 1998 and 2017. The study exploited rich sources of data, including human and animal outbreak data, human demographic information, ecological and land use data, and molecular information for rabies viruses isolated from dogs and wildlife species.

The largest outbreak of human rabies within the study area occurred in Vhembe District in northern Limpopo Province in 2006 was well controlled by intensified central-point dog vaccination campaigns, a community awareness program related to the hazards of dog bites and importance of timely visits to the clinic for post-exposure prophylaxis, education of healthcare workers, and improved availability of vaccine and immuno-globulin, ensuring free of charge for treatment [22]. It was only at this point of the outbreak that veterinary and human rabies professionals started a one-health collaboration given the observation of an increase in both dog and jackal positive cases, and encephalitic cases in hospital admissions in hospitals in this province. Similarly, in Kwazulu-Natal between 2011 and 2014, subsequent to a mass dog vaccination campaign being implemented, a decline in human rabies cases was observed [8]. Even after the successful control of dog and human rabies in 2006, re-emergence of the disease occurred and is supported by cluster 7, demonstrating persistent maintenance of the disease in dog populations. Cases in humans also persisted, and Limpopo Province had the third greatest cases of the country between 2008 and 2018 (*n* = 22), after the Eastern Cape (*n* = 34), and KwaZulu-Natal (*n* = 31) provinces [30]. The majority of human rabies cases in our study were from exposures to infected domestic dogs. This was similar with the figure (75% of human cases were linked to infected domestic dogs) for the entire of South Africa between 2008 and 2018 [30]. Both studies included the cases whose sources were unknown, and rabies caused by wildlife species was rare. Human rabies is extensively underreported (59,000 global deaths estimated versus 26,400 deaths by Global Burden of Disease study based on official records) [44,45]. A recent study conducted in Kwazulu-Natal Province attributed limited hospital-seeking behavior by victims of dog bite injuries to both a lack of awareness of rabies and prevailing poverty in this part of the country [46]. Out of 105 diagnosed human cases between 2008 and 2018 in South Africa, a third (*n* = 32) did not seek medical intervention after exposure [30]. An unknown number of victims might have died at home without being reported, thereby resulting in an underreporting of the disease. Therefore, efforts to better understand rabies epidemiology and transmission dynamics in animal species, as demonstrated in this study, have tremendous merit.

Analyses of spatial scan statistics and phylogenetic relationships of rabies viruses clearly demonstrated the dynamics of animal rabies within the study areas. Previous phylogenetic analyses found the viruses to be very closely related with a mean sequence homology of 97% [12,14] and linked to specific rabies outbreaks in the northern parts of this country [11,18,22,47,48]. Clearly, the rabies viruses analyzed in this study were placed in distinct virus clusters supported by high bootstrap values and clarified the relationships between the historical rabies outbreaks in Limpopo, North-West and Mpumalanga Provinces [11,18,22,47,48]. Further, the data lent support to dogs and black-backed jackals as maintenance host species of the canid rabies biotype [11]. These data further corroborated previous research findings which suggested that the canid rabies viruses of southern Africa have a recent and common progenitor and that this variant was initially introduced with dogs from Angola in the late 1950s and then established cycles in wildlife much later [12,18]. The fact that the dog rabies variant brought into the Republic of South Africa by dogs is now well established in both dogs and wildlife attested to the opportunistic nature of this variant. The dog rabies variant responsible for the human rabies outbreak in 2005–2006 was genetically similar to that of black-backed jackals from southern Zimbabwe [11,22,47] confirming the transboundary nature of rabies. The clusters in principle reflect and confirm the previous rabies outbreaks that were witnessed in the northern provinces of the country in the past two decades. Whilst in Nkomazi and Mbombela (Mpumalanga) and Vhembe (Limpopo) the rabies outbreaks were primarily dog-driven, in the North-West province, there is evidence of involvement of dog and wildlife rabies cycles [11,24]. In Zimbabwe, it was believed that some jackal populations (*C*. *adustus*) do not reach high enough densities to sustain rabies infection cycles in the absence of domestic dogs [49]. Molecular epidemiological analysis indeed confirmed that black-backed jackals in Limpopo [11] and North-West Provinces are capable of sustaining rabies cycles independent of domestic dogs [50]. Rabies virus infection easily crossed species barriers between the dog and wildlife populations (jackals and an African civet cat as examples) in southern Africa [16,50,51].

Group 1B2 variant adapted in jackal populations caused cluster 9 in domestic dogs in 2014 in North West Province. The cluster 9 outbreak in domestic dogs was contained only with 20 dogs, regardless the high *R*_t_ of 3.65, which suggested simultaneous multiple transmission from jackals to dogs. A transmission requires a serial interval, and such rapid transmissions bridging two municipalities may not occur from single infection tree of domestic dogs. During the study period, changes in land use type from grasslands and wooded lands were observed in southern Limpopo and northern North-West Provinces. The North-West Province is also characterized by extensive livestock and crop farming [52]. Jackals prefer open terrain, but they also inhabit woodlands [53] and in such environments can maintain moderate to high density populations in agricultural areas, despite pressure from farmers [11,50,53]. Jackals are highly mobile animals, and their home ranges overlap [53]; therefore, jackal populations can be maintained in areas over a long period as continuous rabies transmission takes place. Dogs in the province roam outside their homesteads, facilitating physical contact with jackals [50,54].

The present study confirmed several rabies clusters (2, 5, 6 and 8) along the KNP. According to our data, there are no human settlements inside the KNP in Limpopo Province, but the staff and families live in the fenced administrative area in Mpumalanga Province. In a private reserve adjacent to the KNP, surveillance of the fence is conducted twice a day, and any dogs observed inside the park are shot and the specimens submitted to the OVR for rabies diagnosis [43]. The dogs include some of which are apparently healthy, which brings detection bias: higher probability of detecting infected dogs may result in the higher number of cases reported than the other areas. The SV BBR E O area encompasses the KNP and surrounding communal livestock farming areas [43], and it is probable that some samples with incomplete records of original location were included in the case count of this municipality, which might result in the detection of clusters. With this surveillance approach, no host shift of rabies has been observed in the KNP [43], and this reduces the risk of spillover from wildlife in the park to domestic dogs. As several rabies variants are prevalent in the surrounding areas, intensive surveillance along the fence must be maintained for the conservation purposes. Rabies transmission for instance to the highly-endangered wild dogs and a spotted hyena in national conservancy areas was reported in the Madikwe Game Reserve (North West Province) in 2014–2015 [24], which can be mitigated through active surveillance.

The basic reproduction number, *R*_0_ is calculated for a totally susceptible population [55]. Accurate rabies vaccination coverage for South Africa is not known, and therefore, we applied the *R*_t_ to quantify the speed of spread. The estimated *R*_0_ for rabies in domestic dogs around the world reportedly varies between 1.05 and 1.72 [38], but the *R*_t_ in some clusters in this study was higher than the expected range. The clusters consisted of different rabies variants (in clusters 5, 7, 8, and 10), and this may point to multiple outbreaks occurring simultaneously. Secondly, clusters 5 and 8 included samples from surveillance and surrounding communities of the KNP, as explained above. Third, in this study an exponential growth fit was attempted, particularly for small outbreaks (clusters 4, 9, and 10), and a sudden increase in sample submissions due to enhanced public awareness about the threat of rabies might be the reason for the high *R*_t_.

Rabies viruses isolated from mongooses were not included in the molecular analyses. The mongoose variant is believed to be indigenous to the Highveld plateau of the Republic of South Africa and was probably introduced into the country at least 200 years ago [56]. Previous antigenic and molecular studies demonstrated that mongoose rabies viruses are different from the canid rabies biotype in jackals [12], as the former is more heterogeneous. In addition, mongoose rabies viruses result in dead-end infections in domestic dogs [13,57]. Although the mongoose rabies viruses exhibit neuro-invasiveness similar to that of the canid rabies biotype, they appear less pathogenic [57]. Therefore, the methodology should not affect our understanding of the epidemiology of rabies and also given that most human rabies are due to the canid rabies biotype.

The ecological risk factor analysis in two time lines with a 10-year interval (of 1998–2002 and 2008–2012) highlighted the areas characterized by woodlands, high temperature, and high precipitation in the driest quarter as high-risk areas. Removal of the data in the KNP did not affect the selection of two predictors: temperature and precipitation, and they were found to be reliable. The risk maps consistently indicated north and northeastern Limpopo Province and northeastern Mpumalanga Province as high risk areas of canine rabies. High precipitation and high temperatures in the driest quarter–winter—offer favorable conditions for subsistence agriculture, for the many rural communities living in these areas. The analysis clarified that high human and dog densities, and urban poverty were not necessarily associated with the risk of canine rabies. *R*_0_ for canine rabies does not depend on dog population density [58]. However, low availability of a susceptible host in dry environment may prevent sustained infection cycle in dogs. While there is a rabies transmission risk between domestic dogs and jackals, the high risk areas of canine rabies did not overlap with the areas from where positive jackal samples frequently originated from. Vaccination of domestic dogs and cats is a legal requirement for pet owners in South Africa [44]. Previous studies reported low dog rabies vaccination rates in low-income areas [9] and among dogs owned by members of minority ethnic groups in Vietnam [29]. The high *R*_t_ in several clusters suggested that dog rabies vaccination coverage was very low in these areas including the extensive rural areas. Intensive dog vaccination campaigns were carried out in Limpopo Province in 2006 [22], but given these are not sustainable, re-emergence of rabies in dogs was observed. This and other studies have confirmed that rabies variants are pivotal in rabies outbreaks in the northern parts of South Africa. Control efforts should be focused on improving the geographical distribution of dog rabies vaccination coverage.

This study has a number of limitations. A large number of positive animal rabies cases lacked information of exact locations of origin, and therefore aggregated data at the municipality level were used for the analyses. This method is commonly used in spatial epidemiology [59], but data aggregation has a risk of missing accurate socio-economic and ecological associations in a local context. The lack of accurate dog population data and vaccination records was another challenge in assessing rabies risks. It is highly recommended to establish a monitoring system for rabies cases with geographical locations, dog population, and dog vaccination.

This study enhanced current understanding of the dynamics of rabies transmission in humans and animals in northern South Africa over a 20-year period and identified high-risk areas and ecological conditions that may affect the transmission dynamics. The results will be useful to identify hot spots and this could be useful for planning targeted rabies control programs in dogs thereby reducing human rabies cases, which would contribute positively to achieving zero human deaths by 2030 in South Africa and the region at large.

## Supporting information

S1 Table Viruses used in this study. (DOCX)

S2 Table Principal components of land cover data for 2001 in the dataset including the Kruger National Park. (DOCX)

S3 Table Principal components of land cover data for 2001 in the dataset excluding the Kruger National Park. (DOCX)

S4 Table A comparison of multivariable analysis results for INLA using a zero-inflated convolution model with negative binomial errors for predicting dog rabies cases between 1998 and 2002 including the Kruger National Park. (DOCX)

S5 Table A comparison of multivariable analysis results for INLA using a zero-inflated convolution model with negative binomial errors for predicting dog rabies cases between 1998 and 2002 excluding the Kruger National Park. (DOCX)

S6 Table Principal components of land cover data for 2011 in the dataset including the Kruger National Park. (DOCX)

S7 Table Principal components of land cover data for 2011 in the dataset excluding the Kruger National Park. (DOCX)

S8 Table Multivariable analysis results for INLA using a zero-inflated convolution model with negative binomial errors for dog rabies cases between 2008 and 2012 using the dataset including KNP. (DOCX)

S9 Table A comparison of multivariable analysis results for INLA using a zero-inflated convolution model with negative binomial errors for dog rabies cases between 2008 and 2012 excluding the KNP. (DOCX)

S1 Fig Map of study area with land cover classification used in the analysis. < https://globalmaps.github.io/glcnmo.html>
(TIF)

S2 Fig Map of study area with human footprint of years 1995–2004. https://sedac.ciesin.columbia.edu/data/set/wildareas-v2-last-of-the-wild-geographic. SEDAC Data Licenses: https://sedac.ciesin.columbia.edu/data-submission
(TIF)

S3 Fig Map of study area with human footprint of year 2009. https://sedac.ciesin.columbia.edu/data/set/wildareas-v3-2009-human-footprint SEDAC Data Licenses: https://sedac.ciesin.columbia.edu/data-submission
(TIF)
