# Supplementary material for: Spatio-temporal epidemiology of animal and human rabies in northern South Africa between 1998 and 2017
Source: PLoS Negl Trop Dis. 2022 Jul 29;16(7):e0010464. doi: 10.1371/journal.pntd.0010464 (PMC9365189; doi:10.1371/journal.pntd.0010464)
Supplement: S1 Table — (DOCX) [file pntd.0010464.s001.docx]

**Supplementary Table 1. Viruses used in this study**

| **Virus reference #** | **Locality of origin** | **Genbank accession number** | **Reference** |
| --- | --- | --- | --- |
| 306/1998 | Warmbad | AF177105 | Zulu et al., 2007 [4] |
| 208/1999 | Waterberg | EF686061 | Zulu et al., 2007 [4] |
| 191/2001 | Soutpansberg | EF686073 | Zulu et al., 2007 [4] |
| 42/2001 | Musina | EF686067 | Zulu et al., 2007 [4] |
| 207/2000 | Potgietersrus | EF686063 | Zulu et al., 2007 [4] |
| 294/2006 | Louis Trichardt | EF686137 | Zulu et al., 2007 [4] |
| 914/2006 | Mara, Makhado | OL790407 | This study |
| 819/2005 | Soutpansberg | EF686121 | Zulu et al., 2007 [4] |
| 130/2015 | Musina | MK098243 | Schepers C., 2018 [3] |
| 201/2017 | Bontveld, Makhado | OL790408 | This study |
| 391/2015 | Hoedspruit | MK098238 | Schepers C., 2018 [3] |
| 475/2015 | Hoedspruit | MK098240 | Schepers C., 2018 [3] |
| 149/2015 | Orpen | MK103221 | Schepers C., 2018 [3] |
| 256/2013 | Charleston, Bushbuckridge | MK103228 | Schepers C., 2018 [3] |
| 1237/2010 | Ngongane, Nelspruit | OL790409 | This study |
| 576/2008 | Mbombela | FJ842724 | Mkhize et al., 2010 [1] |
| 1157/2008 | Kruger National Park South | FJ842733 | Mkhize et al., 2010 [1] |
| 27/2001 | Barberton | EF686096 | Zulu et al., 2007 [4] |
| 344/2000 | Barberton | EF686103 | Zulu et al., 2007 [4] |
| 390/2000 | Barberton | EF686079 | Zulu et al., 2007 [4] |
| 221/2006 | Nkomazi | EF686146 | Zulu et al., 2007 [4] |
| 136/2002 | Piet Retief | EF686101 | Zulu et al., 2007 [4] |
| 172/2015 | Olifantsvlei, Lerato | OL790410 | This study |
| 520/2014 | Lerato | OL790411 | This study |
| 380/2014 | Vlakte, Swartruggens | OL790412 | This study |
| 609/2014 | Lerato | MK103306 | Schepers C., 2018 [3] |
| 519/2014 | Veeplaas, Lerato | MK103304 | Schepers C., 2018 [3] |
| 491/2014 | Lerato | OL790413 | This study |
| 343/2014 | Shimunini | MK098234 | Schepers C., 2018 [3] |
| 84/2006 | Sibasa | EF686123 | Zulu et al., 2007 [4] |
| 189/2007 | Sibasa | GU808520 | Sabeta et al., 2011 [2] |
| 53/2013 | Mutale | MK098242 | Schepers C., 2018 [3] |
| 171/2014 | Dzanani village | MK098258 | Schepers C., 2018 [3] |
| 87/2016 | Alan Burman, Tzaneen | MK098250 | Schepers C., 2018 [3] |
| 1069/2016 | Loschof, Tzaneen | MK098257 | Schepers C., 2018 [3] |
| 1034/2006 | Tzaneen | HM060258 | Sabeta et al., 2011 [2] |

**Reference**

1. Mkhize GC, Ngoepe CE, Du Plessis BJA, Reininghaus B, Sabeta CT. 2009. Re-emergence of dog rabies in Mpumalanga Province, South Africa. Vector Borne Zoonotic Dis. 10 (9): 921-926.

2. Sabeta C, Mkhize GC, Ngoepe EC. 2011. An evaluation of dog rabies control in Limpopo province (South Africa). Epidemiol. Infect. 139 (10): 1470 – 1475.

3. Schepers C. 2018. Molecular epidemiology of rabies in domestic animals and wildlife in South Africa. Unpublished data.

4. Zulu GC, Sabeta CT, Nel LH. 2007. Molecular epidemiology of rabies: focus on domestic dogs (*Canis familiaris*) and black-backed jackals (*Canis mesomelas*) from northern South Africa. Virus Res. 140 (1-2): 71-78.
