# Supplementary material for: Spatio-temporal epidemiology of animal and human rabies in northern South Africa between 1998 and 2017
Source: PLoS Negl Trop Dis. 2022 Jul 29;16(7):e0010464. doi: 10.1371/journal.pntd.0010464 (PMC9365189; doi:10.1371/journal.pntd.0010464)
Supplement: S2 Table — (DOCX) [file pntd.0010464.s002.docx]

Supplementary Table 2. Principal components of land cover data for 2001 in the dataset including the Kruger National Park.

| Land type | PC1 | PC2 | PC3 | PC4 | PC5 |
| --- | --- | --- | --- | --- | --- |
| Woodland | 0.409 | -0.533 | 0.285 | 0.477 | -0.082 |
| Shrub | -0.721 | 0.029 | 0.058 | -0.087 | -0.142 |
| Herbaceous | 0.534 | 0.391 | 0.009 | -0.527 | 0.135 |
| Farmland | 0.037 | 0.112 | -0.817 | 0.408 | 0.318 |
| Bare | -0.107 | 0.449 | 0.497 | 0.398 | 0.618 |
| Urban | 0.126 | 0.590 | 0.025 | 0.404 | -0.687 |
| Standard deviation | 1.369 | 1.173 | 1.067 | 0.934 | 0.859 |
| Proportion of variance | 0.313 | 0.229 | 0.190 | 0.145 | 0.123 |
| Cumulative proportion of variance | 0.313 | 0.542 | 0.732 | 0.877 | 1.000 |
