# Supplementary material for: Spatio-temporal epidemiology of animal and human rabies in northern South Africa between 1998 and 2017
Source: PLoS Negl Trop Dis. 2022 Jul 29;16(7):e0010464. doi: 10.1371/journal.pntd.0010464 (PMC9365189; doi:10.1371/journal.pntd.0010464)
Supplement: S3 Table — (DOCX) [file pntd.0010464.s003.docx]

Supplementary Table 3. Principal components of land cover data for 2001 in the dataset excluding the Kruger National Park.

| Land type | PC1 | PC2 | PC3 | PC4 | PC5 |
| --- | --- | --- | --- | --- | --- |
| Woodland | 0.448 | -0.623 | -0.299 | -0.296 | -0.484 |
| Shrub | -0.721 | -0.046 | -0.065 | 0.163 | -0.668 |
| Herbaceous | 0.508 | 0.617 | 0.013 | 0.304 | -0.518 |
| Farmland | 0.014 | 0.040 | 0.821 | -0.523 | -0.225 |
| Bare | -0.143 | 0.476 | -0.482 | -0.721 | -0.008 |
| Urban | 0.116 | 0.595 | 0.000 | 0.417 | -0.678 |
| Standard deviation | 1.366 | 1.077 | 1.069 | 0.912 | 0.016 |
| Proportion of variance | 0.373 | 0.232 | 0.229 | 0.167 | 0.000 |
| Cumulative proportion of variance | 0.373 | 0.605 | 0.833 | 1.000 | 1.000 |
