# Supplementary material for: Spatio-temporal epidemiology of animal and human rabies in northern South Africa between 1998 and 2017
Source: PLoS Negl Trop Dis. 2022 Jul 29;16(7):e0010464. doi: 10.1371/journal.pntd.0010464 (PMC9365189; doi:10.1371/journal.pntd.0010464)
Supplement: S4 Table — (DOCX) [file pntd.0010464.s004.docx]

Supplementary Table 4. A comparison of multivariable analysis results for INLA using a zero-inflated convolution model with negative binomial errors for predicting dog rabies cases between 1998 and 2002 including the Kruger National Park.

| Models | PC2 | BIO17 | Spatially structured residual | Non-structured residual | DIC | WAIC |
| --- | --- | --- | --- | --- | --- | --- |
| Purely spatial | - | - | 1822.8 | 1854.1 | 166.5 | 167.3 |
| PC2 | -0.498 | - | 1850.0 | 1882.7 | 163.4 | 165.2 |
| BIO17 | - | 0.058 | 1946.0 | 2033.4 | 163.1 | 164.3 |
| **PC2+ BIO17** | **-0.336** | **0.042** | **1877.4** | **1907.1** | **162.4** | **163.9** |
