# Supplementary material for: Spatio-temporal epidemiology of animal and human rabies in northern South Africa between 1998 and 2017
Source: PLoS Negl Trop Dis. 2022 Jul 29;16(7):e0010464. doi: 10.1371/journal.pntd.0010464 (PMC9365189; doi:10.1371/journal.pntd.0010464)
Supplement: S5 Table — (DOCX) [file pntd.0010464.s005.docx]

Supplementary Table 5. A comparison of multivariable analysis results for INLA using a zero-inflated convolution model with negative binomial errors for predicting dog rabies cases between 1998 and 2002 excluding the Kruger National Park.

| Models | PC2 | BIO17 | Spatially structured residual | Non-structured residual | DIC | WAIC |
| --- | --- | --- | --- | --- | --- | --- |
| Purely spatial | - | - | 2503.1 | 2255.3 | 156.1 | 157.3 |
| PC2 | -0.513 | - | 2385.3 | 2465.8 | 152.3 | 154.3 |
| BIO17 | - | 0.061 | 1840.1 | 1847.3 | 151.8 | 153.6 |
| **PC2+ BIO17** | **-0.321** | **0.042** | **2204.7** | **2339.2** | **151.1** | **153.1** |
