# Supplementary material for: Spatio-temporal epidemiology of animal and human rabies in northern South Africa between 1998 and 2017
Source: PLoS Negl Trop Dis. 2022 Jul 29;16(7):e0010464. doi: 10.1371/journal.pntd.0010464 (PMC9365189; doi:10.1371/journal.pntd.0010464)
Supplement: S6 Table — (DOCX) [file pntd.0010464.s006.docx]

Supplementary Table 6. Principal components of land cover data for 2011 in the dataset including the Kruger National Park.

| Land type | PC1 | PC2 | PC3 | PC4 | PC5 | PC6 |
| --- | --- | --- | --- | --- | --- | --- |
| Woodland | -0.403 | -0.536 | 0.218 | 0.530 | -0.005 | -0.471 |
| Shrub | 0.708 | -0.027 | 0.087 | -0.117 | -0.185 | -0.665 |
| Herbaceous | -0.547 | 0.371 | 0.033 | -0.522 | 0.097 | -0.529 |
| Farmland | 0.037 | 0.264 | -0.771 | 0.405 | 0.339 | -0.238 |
| Bare | 0.153 | 0.393 | 0.573 | 0.279 | 0.644 | -0.006 |

| Urban | -0.108 | 0.592 | 0.144 | 0.437 | -0.653 | -0.015 |
| --- | --- | --- | --- | --- | --- | --- |
| Standard deviation | 1.379 | 1.177 | 1.069 | 0.921 | 0.849 | 0.009 |

| Proportion of variance | 0.317 | 0.231 | 0.191 | 0.141 | 0.120 | 0.000 |
| --- | --- | --- | --- | --- | --- | --- |
| Cumulative proportion of variance | 0.317 | 0.548 | 0.738 | 0.880 | 1.000 | 1.000 |
