# Supplementary material for: Spatio-temporal epidemiology of animal and human rabies in northern South Africa between 1998 and 2017
Source: PLoS Negl Trop Dis. 2022 Jul 29;16(7):e0010464. doi: 10.1371/journal.pntd.0010464 (PMC9365189; doi:10.1371/journal.pntd.0010464)
Supplement: S7 Table — (DOCX) [file pntd.0010464.s007.docx]

Supplementary Table 7. Principal components of land cover data for 2011 in the dataset excluding the Kruger National Park.

| Land type | PC1 | PC2 | PC3 | PC4 | PC5 | PC6 |
| --- | --- | --- | --- | --- | --- | --- |
| Woodland | -0.423 | 0.465 | -0.264 | 0.580 | 0.062 | -0.441 |
| Shrub | 0.691 | 0.029 | -0.140 | -0.117 | 0.172 | -0.677 |
| Herbaceous | -0.558 | -0.330 | 0.031 | -0.521 | -0.140 | -0.536 |
| Farmland | 0.077 | -0.146 | 0.815 | 0.392 | -0.311 | -0.243 |
| Bare | 0.127 | -0.484 | -0.496 | 0.318 | -0.635 | -0.006 |
| Urban | -0.100 | -0.647 | -0.025 | 0.351 | 0.669 | -0.015 |

| Standard deviation | 1.405 | 1.146 | 1.071 | 0.912 | 0.855 | 0.010 |
| --- | --- | --- | --- | --- | --- | --- |

| Proportion of variance | 0.329 | 0.219 | 0.191 | 0.139 | 0.122 | 0.000 |
| --- | --- | --- | --- | --- | --- | --- |
| Cumulative proportion of variance | 0.329 | 0.548 | 0.739 | 0.878 | 1.000 | 1.000 |
