# Supplementary material for: Spatio-temporal epidemiology of animal and human rabies in northern South Africa between 1998 and 2017
Source: PLoS Negl Trop Dis. 2022 Jul 29;16(7):e0010464. doi: 10.1371/journal.pntd.0010464 (PMC9365189; doi:10.1371/journal.pntd.0010464)
Supplement: S8 Table — (DOCX) [file pntd.0010464.s008.docx]

Supplementary Table 8. Multivariable analysis results for INLA using a zero-inflated convolution model with negative binomial errors for dog rabies cases between 2008 and 2012 using the dataset including KNP.

| Models | Dog population | PC2 | BIO9 | BIO17 | Spatially structured residual | Non-structured residual | DIC | WAIC |
| --- | --- | --- | --- | --- | --- | --- | --- | --- |
| Purely spatial | - | - | - | - | 1833.6 | 1880.4 | 280.7 | 283.3 |
| Dog population | 0.173 | - | - | - | 1826.8 | 1878.0 | 273.4 | 275.9 |
| PC2 | - | -0.878 | - | - | 1843.1 | 1888.1 | 269.1 | 272.7 |
| BIO9 | - | - | 0.385 | - | 1840.7 | 1883.7 | 268.6 | 272.6 |
| BIO17 | - |  | - | 0.104 | 1813.0 | 1873.5 | 271.1 | 276.6 |
| Dog population + PC2 | 0.116 | -0.824 | - | - | 1841.7 | 1894.1 | 264.8 | 267.7 |
| Dog population + BIO9 | 0.111 | - | 0.258 | - | 1846.0 | 1908.1 | 267.5 | 271.4 |
| Dog population + BIO17 | 0.095 | - | - | 0.058 | 1806.2 | 1896.8 | 272.0 | 277.6 |
| PC2 + BIO9 | - | -0.509 | 0.233 | - | 1826.0 | 1883.0 | 251.8 | 258.3 |
| PC2 + BIO17 | - | -0.755 | - | 0.082 | 1860.5 | 1920.0 | 264.0 | 269.2 |
| **BIO9 + BIO17** | **-** | **-** | **0.417** | **0.124** | **1858.3** | **1904.7** | **251.7** | **257.1** |
| Dog population + PC2 + BIO9 | 0.102 | -0.560 | 0.139 | - | 1842.9 | 1906.3 | 265.2 | 270.2 |
| Dog population + PC2 + BIO17 | 0.077 | -0.788 | - | 0.046 | 1909.1 | 1989.9 | 264.4 | 270.4 |
| PC2 + BIO9 +BIO17 |  | 0.110 | 0.452 | 0.132 | 1847.0 | 1886.2 | 253.4 | 259.5 |
| Dog population + PC2 + BIO9 + BIO17 | -0.026 | 0.208 | 0.497 | 0.147 | 1896.8 | 1847.1 | 254.9 | 261.5 |
