# Supplementary material for: Spatio-temporal epidemiology of animal and human rabies in northern South Africa between 1998 and 2017
Source: PLoS Negl Trop Dis. 2022 Jul 29;16(7):e0010464. doi: 10.1371/journal.pntd.0010464 (PMC9365189; doi:10.1371/journal.pntd.0010464)
Supplement: S9 Table — (DOCX) [file pntd.0010464.s009.docx]

Supplementary Table 9. A comparison of multivariable analysis results for INLA using a zero-inflated convolution model with negative binomial errors for dog rabies cases between 2008 and 2012 excluding the KNP.

| Models | Dog population | PC2 | BIO9 | BIO17 | Spatially structured residual | Non-structured residual | DIC | WAIC |
| --- | --- | --- | --- | --- | --- | --- | --- | --- |
| Purely spatial | - | - | - | - | 1929.5 | 1914.4 | 202.5 | 203.5 |
| Dog population | 0.116 | - | - | - | 2084.6 | 1850.0 | 198.9 | 201.4 |
| PC2 | - | 0.352 | - | - | 2024.1 | 2059.8 | 203.3 | 205.9 |
| BIO9 (Temp.) | - | - | 0.296 | - | 1797.7 | 1846.0 | 198.5 | 201.0 |
| BIO17 (Precip.) | - |  | - | 0.045 | 1740.6 | 1840.6 | 202.3 | 206.7 |
| Dog population + PC2 | 0.114 | 0.376 | - | - | 2057.9 | 2128.9 | 199.5 | 202.6 |
| Dog population + BIO9 | 0.089 | - | 0.207 | - | 1833.5 | 1938.4 | 197.3 | 201.3 |
| Dog population + BIO17 | 0.116 | - | - | 0.000 | 1815.8 | 1908.3 | 200.5 | 205.3 |
| PC2 + BIO9 | - | -0.123 | 0.328 | - | 1745.7 | 1869.1 | 199.9 | 202.8 |
| PC2 + BIO17 | - | 0.281 | - | 0.041 | 1867.4 | 1829.7 | 203.6 | 208.9 |
| BIO9 + BIO17 | - | - | 0.385 | 0.085 | 2012.6 | 2206.6 | 194.2 | 199.7 |
| Dog population + PC2 + BIO9 | 0.093 | 0.028 | 0.197 | - | 1983.1 | 1997.0 | 199.0 | 203.8 |
| Dog population + PC2 + BIO17 | 0.120 | 0.397 | - | -0.008 | 2485.3 | 2695.8 | 201.3 | 206.8 |
| PC2 + BIO9 +BIO17 |  | -0.990 | 0.644 | 0.137 | 1897.3 | 1851.984 | 191.0 | 195.9 |
| **Dog population + PC2 + BIO9 + BIO17** | **-0.196** | **-2.189** | **1.171** | **0.277** | **1866.7** | **1764.2** | **189.7** | **193.6** |
