# Supplementary figures and images for: Spatio-temporal epidemiology of animal and human rabies in northern South Africa between 1998 and 2017
Source: PLoS Negl Trop Dis. 2022 Jul 29;16(7):e0010464. doi: 10.1371/journal.pntd.0010464 (PMC9365189; doi:10.1371/journal.pntd.0010464)

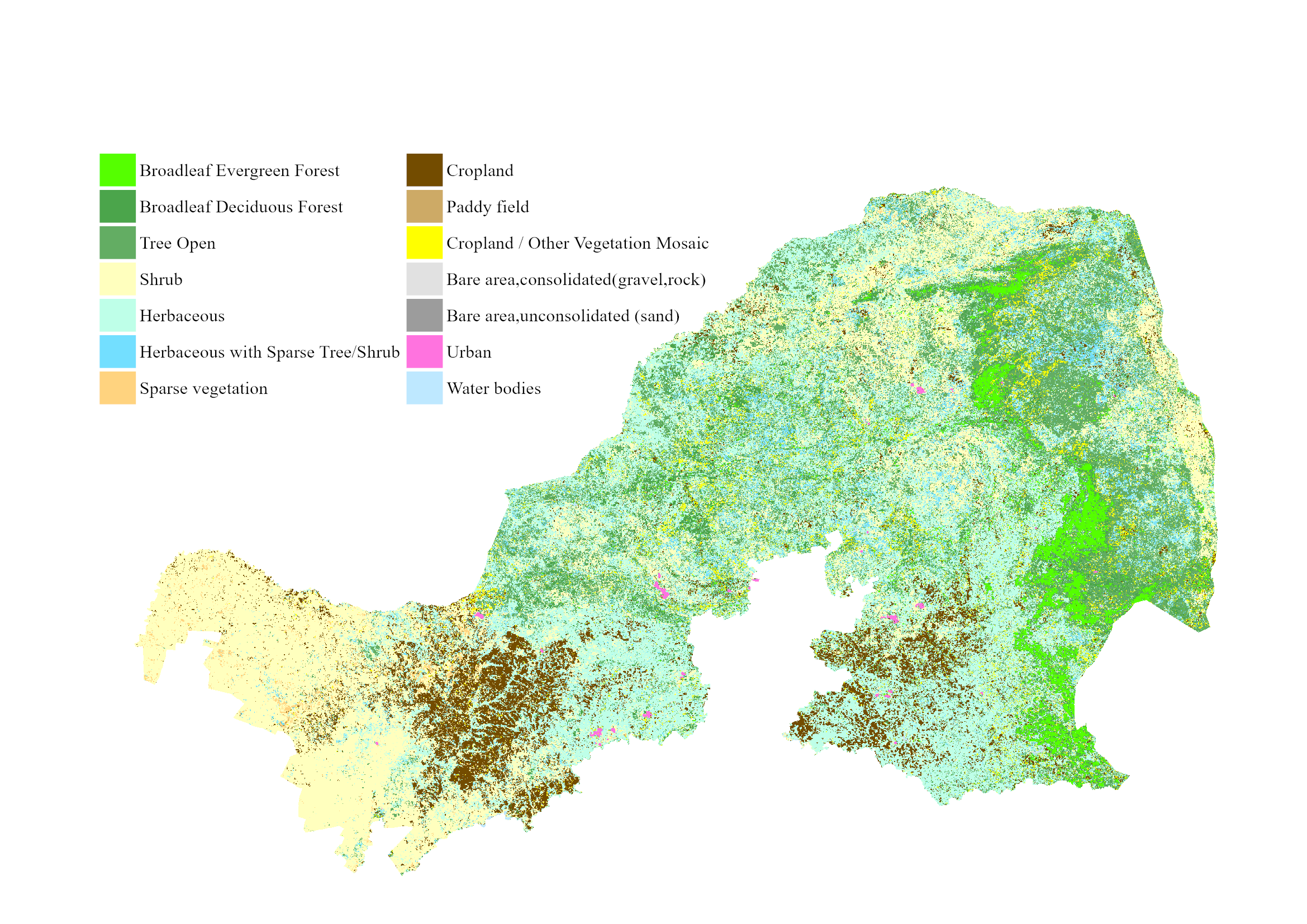

Supplement: S1 Fig — < https://globalmaps.github.io/glcnmo.html> (TIF) [file pntd.0010464.s010.tif]

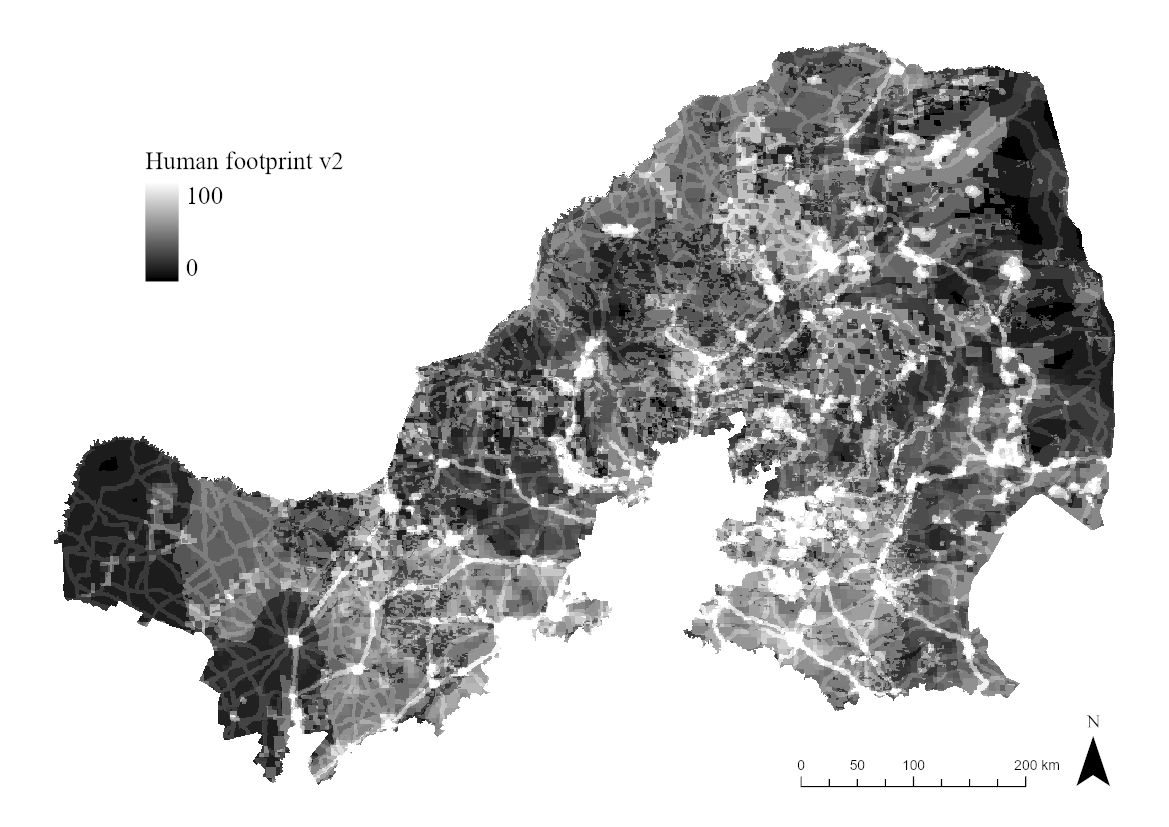

Supplement: S2 Fig — https://sedac.ciesin.columbia.edu/data/set/wildareas-v2-last-of-the-wild-geographic. SEDAC Data Licenses: https://sedac.ciesin.columbia.edu/data-submission (TIF) [file pntd.0010464.s011.tif]

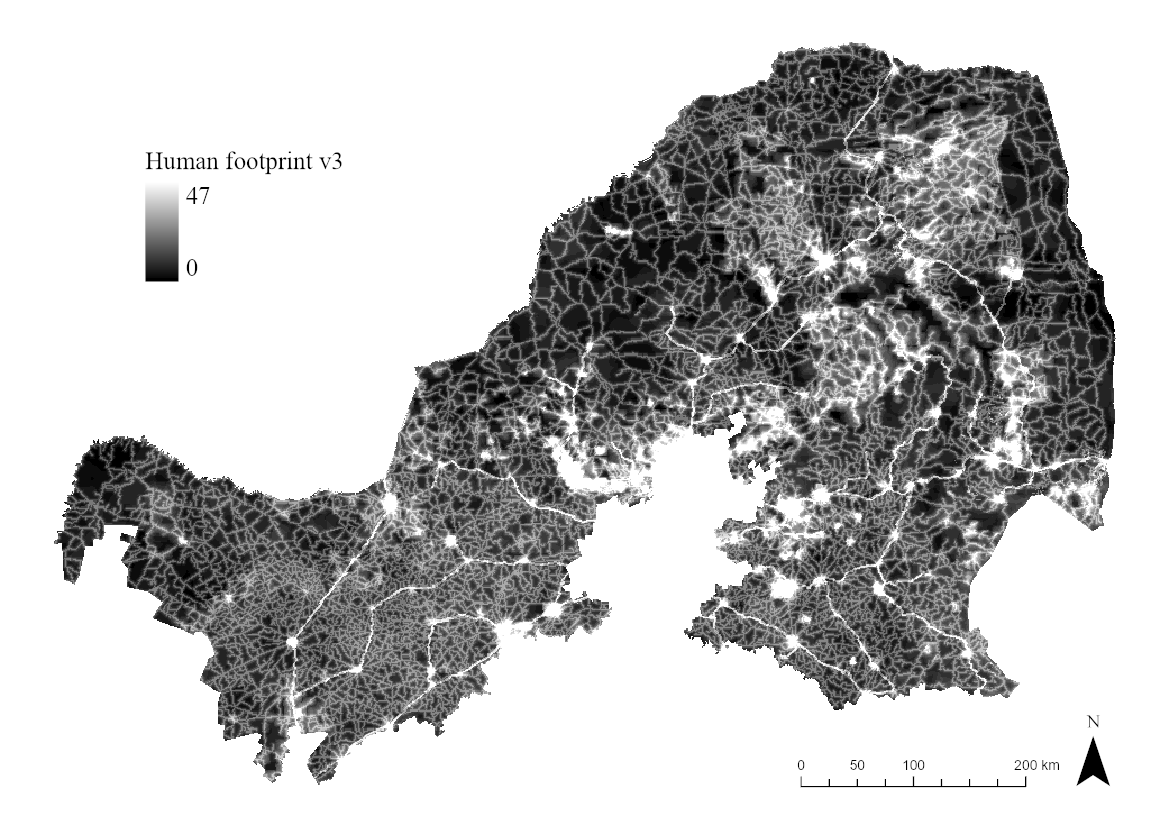

Supplement: S3 Fig — https://sedac.ciesin.columbia.edu/data/set/wildareas-v3-2009-human-footprint SEDAC Data Licenses: https://sedac.ciesin.columbia.edu/data-submission (TIF) [file pntd.0010464.s012.tif]
